# Association between urinary polycyclic aromatic hydrocarbon metabolites and diabetes-related mortality: a longitudinal study in U.S. adults

**DOI:** 10.1016/j.clinsp.2026.101169

**Published:** 2026-09-29

**Authors:** Ao Li, Yuxin Pan, Mingcong Chen, Yang Meng, Yang Zhang, Mingyi Zhao, Lin Liu

**Affiliations:** Department of pediatrics, The Third Xiangya Hospital of Central South University, Changsha Hunan, China

**Keywords:** Polycyclic aromatic hydrocarbons, NHANEs, All-cause mortality among adults with diabetes, WQS, Diabetes

## Abstract

**Introduction:**

This study aimed to explore the association between urinary Polycyclic Aromatic Hydrocarbons (PAHs) and diabetes prevalence and all-cause mortality in U.S. adults using data from 8,124 participants in the 2003–2014 NHANES cycles

**Methods:**

Weighted logistic regression and Cox proportional hazards models were employed to analyze the effects of individual and combined PAH metabolites. Confounders were adjusted, and nonlinear relationships were explored through restricted cubic splines.

**Results:**

Weighted quantile sum regression revealed that co-exposure to six urinary PAHs was significantly associated with higher diabetes prevalence (OR = 1.131, 95% CI 1.008–1.269) and all-cause mortality (OR = 1.330, 95% CI 1.051–1.684), with 2-hydroxynaphthalene contributing most to diabetes risk and 1-hydroxynaphthalene to mortality risk within the mixture. In exploratory analyses of individual metabolites, nominal (unadjusted for multiple comparisons) positive associations were observed for ΣPAHs and 1-hydroxynaphthalene with diabetes prevalence. ΣPAHs also showed a modest association with all-cause mortality.

**Conclusions:**

These findings suggest that PAH exposure is associated with a higher prevalence of diabetes and, among individuals with diabetes, higher all-cause mortality.

## Highlights

• Urinary PAHs correlate positively with diabetes prevalence and all-cause mortality.
• 2-Hydroxynaphthalene has the strongest association with diabetes prevalence.
• 1-Hydroxynaphthalene most closely associates with all-cause mortality in diabetics.
• A nonlinear relationship exists between 2-hydroxynaphthalene and diabetic mortality.

## Graphical abstract

**Figure:**
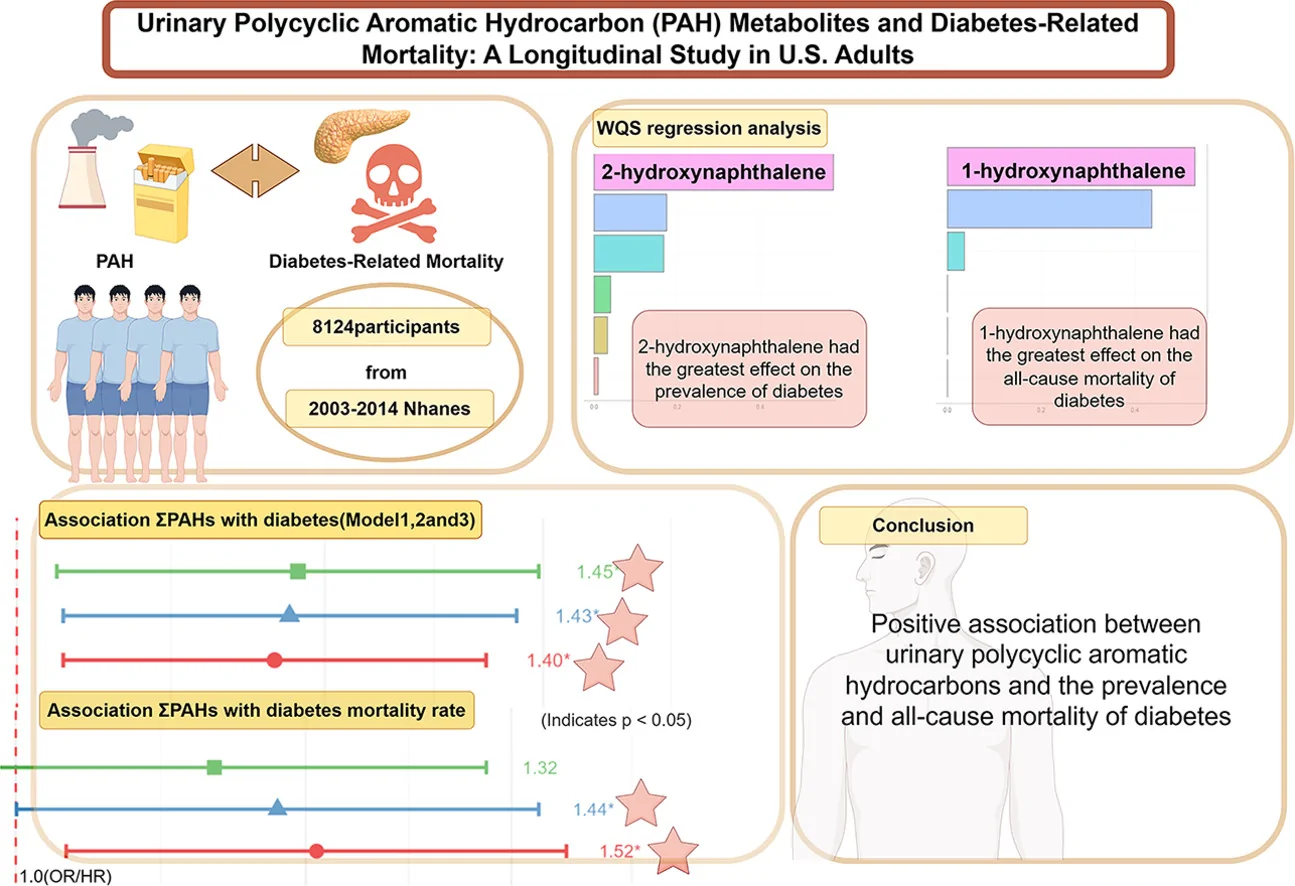

## Introduction

Polycyclic Aromatic Hydrocarbons (PAHs) are a large class of organic compounds containing two or more fused benzene rings arranged in various configurations. They are characterized by chemical stability, low volatility, and low water solubility^1^ PAHs are widespread environmental contaminants formed as a result of incomplete combustion of organic materials, such as fossil fuels^2^ The pollution primarily stems from partial combustion of vehicle exhausts, asphalt fumes, industrial exhaust, and waste incineration. This makes PAHs an unavoidable pollutant in human daily life^3^ The potential harm of PAHs to human health has attracted widespread attention. Studies have shown that these compounds are carcinogenic, teratogenic, and genotoxic and damage the immune system; they can cause various health problems, including rheumatoid arthritis,^4^ non-alcoholic fatty liver disease,^5^ and chronic kidney disease^6^ by inducing pro-inflammatory cytokines. In addition, total PAHs (∑PAHs), including eight urinary hydroxylated PAH metabolites from four parent compounds (naphthalene, fluorene, phenanthrene, pyrene), are significantly associated with higher all-cause mortality and cancer-specific mortality^7^ The National Institute for Occupational Safety and Health (NIOSH) recommends that workplace airborne PAH exposure should be below 0.1 mg/m^3^. However, industrial development has led to long-term exposure to environments exceeding this standard. Long-term exposure to PAHs is significantly correlated with various diseases and even death.

Diabetes, a chronic disease, profoundly influences human health and quality of life. The global prevalence of diabetes in 2019 was estimated at 9.3% (about 463 million people), which is expected to rise to 10.2% (about 578 million people) by 2030 and may further climb to 10.9% (about 700 million people) by 2045^8^ Previous studies have revealed a significant association between PAHs and diabetes. A study on the general population in the United States showed that high PAH exposure levels were positively associated with diabetes, and this association would be influenced by Body Mass Index (BMI)^9^ In addition, a meta-analysis concluded that urinary PAH metabolites may have adverse effects on type 2 diabetes^10^ More specifically, a study analyzed the Odds Ratio (OR) of individual PAH metabolites for diabetes risk through logistic regression model analysis, with the lowest quartile of PAH exposure as a reference. The results showed that 1- and 2-hydroxynaphthalene, 2-hydroxyphenanthrene, and total urinary PAH biomarkers were significantly associated with the risk of diabetes in adults aged 20‒65 years in the United States^11^ However, most current studies only focus on a single type of PAH, while ignoring the combined effect of PAHs on the prevalence of diabetes. In addition, the potential correlation of single PAH and their mixtures with all-cause mortality of patients with diabetes has not been thoroughly explored in existing studies.

Based on six cycles of data from the NHANES database between 2003 and 2014, this study analyzed 8,124 participants. With its large sample size and broad representativeness, this study provided reliable foundational data. Since previous studies have seldom examined the combined effects of PAH mixtures, the Weighted Quantile Sum (WQS) model was applied in this study to systematically evaluate the overall association of six mixtures of PAH metabolites with diabetes prevalence and all-cause mortality. This study aimed to clarify the overall effect of PAH exposure and identify key PAH types, so as to determine the long-term hazards of environmental exposure to diabetes patients. Through the analysis of urinary PAH metabolites, we provided new scientific evidence for assessing the association between PAH exposure and diabetes prevalence as well as all-cause mortality. This method not only fills the gaps in existing research but also provides important evidence for identifying potential hazards of environmental pollutants to public health. Moreover, the research can provide data support for the early intervention of diabetes and personalized health management strategies, thereby providing practical guidance for reducing the burden of diabetes.

## Materials and methods

### Study population and design

The data were derived from the NHANES database. This database encompasses the health and nutritional status of adults and children in the USA using the stratified, multistage probability cluster sampling method. Continuous NHANES is a series of surveys conducted every two years since 1999, involving about 10000 participants for each period. It collects data through physical examinations, interviews, and laboratory tests. In addition, the NHANES has collected data on deaths in the population from 2003 to 2014. Urinary PAHs were measured in one-third of people aged ≥6-years. Data from six cycles of the NHANES 2003‒2014 were used, including 61087 subjects. We excluded 46135 participants with missing data on urinary PAHs, 4435 participants with missing data on mortality, 219 participants with missing data on diabetes, and 2174 participants with missing data on covariates. Finally, 8124 participants were included in this cohort study (Fig. 1). The NHANES database has been approved by the Ethics Review Board of the National Center for Health Statistics, and participants have provided written informed consent forms to participate in surveys.

**Fig. 1:**
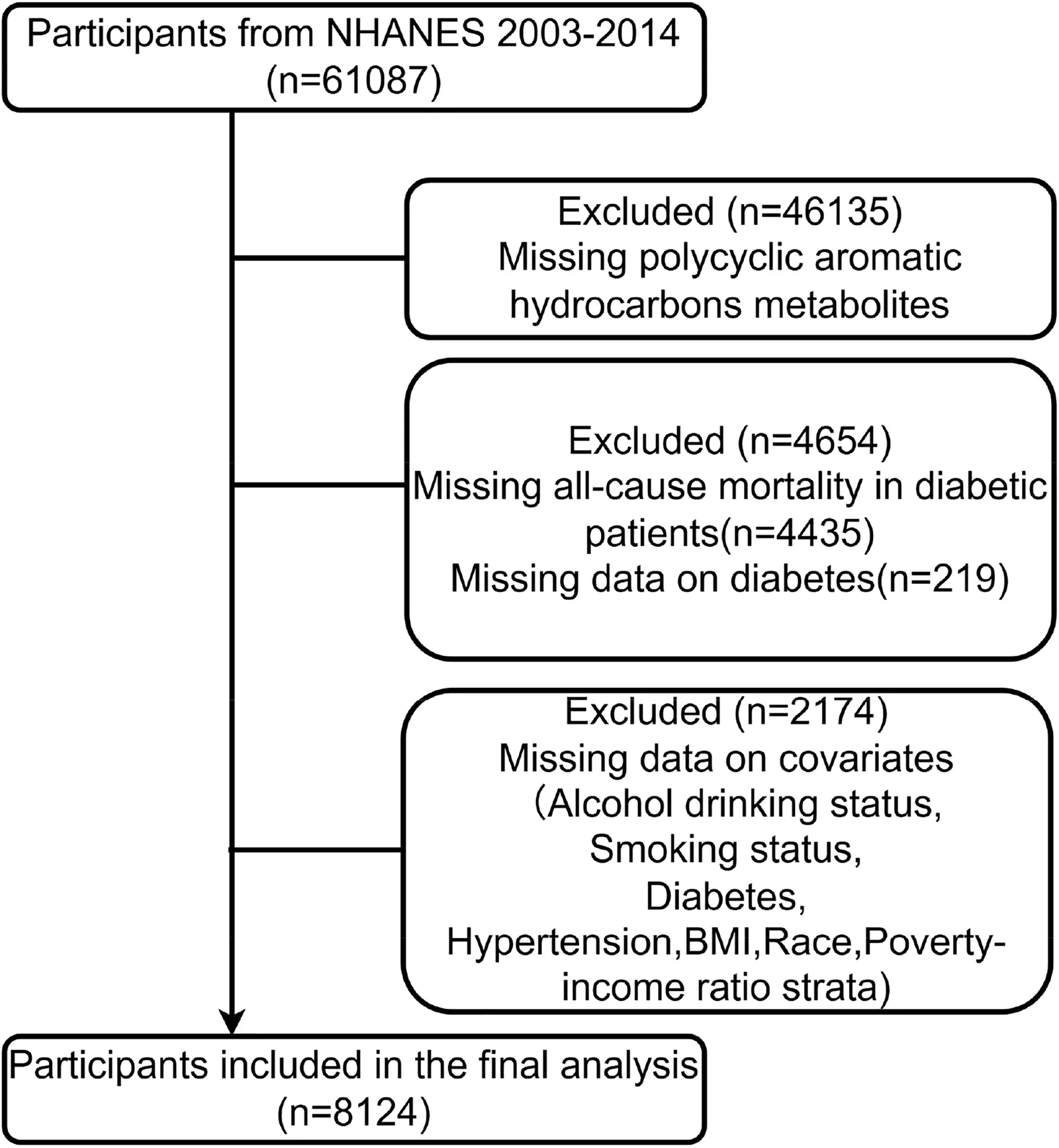
Data processing flowchart.

### Measurement of urinary PAH metabolites and their associations

Urinary PAH metabolites are considered stable biomarkers of PAH exposure via multiple routes or pathways. Urine samples were collected at the Mobile Examination Center, stored at -20°C, and sent (for analysis) to the U.S. CDC’s National Center for Environmental Health. The analytical procedure included enzymatic hydrolysis of glucose in urine to produce its aldose/sulfate derivatives, Solid-Phase Extraction (SPE), and separation and quantification using isotope dilution High-Performance Liquid Chromatography (HPLC)-tandem Mass Spectrometry (online SPE-HPLC-MS/MS). Given differences in urinary PAH metabolites measured in different cycles, this study utilized data from the six cycles to investigate the content of 6 urinary PAHs: 1-hydroxynapthalene, 2-hydroxynapthalene, 3-hydroxyfluorene, 2-hydroxyfluorene, 1-hydroxyphenanthrene, and 1-hydroxypyrene.

### Definition and mortality of diabetes

Diabetes was defined based on self-reported physician diagnosis: “Have you ever been told by a doctor or other health professional that you have diabetes?” If the participant answered “yes”, she/he was classified as having diabetes. Mortality data were sourced from the public National Death Index to determine the mortality status and cause of death until December 31, 2018. In this study, the final outcomes were the prevalence and all-cause mortality among adults with diabetes.

### Covariates

Participant demographics included age, sex (male/female), race (non-Hispanic whites, non-Hispanic blacks, Mexican Americans, other Hispanics, or other races), and poverty-income ratio (PIR, < 1.5, 1.5‒3.5, > 3.5). Hypertension was defined as self-reported hypertension. Participants were considered drinkers if they consumed at least 12-drinks in any year. BMI was calculated as weight in kilograms divided by height in meters squared (kg/m^2^). Participants were classified as underweight (BMI < 18.5), normal weight (BMI = 18.5‒25), overweight (BMI = 25‒30), and obese (BMI > 30).

### Statistical analysis

Statistical analysis was performed using the complex sampling design and weights recommended by NHANES to calculate the weighted average for continuous variables and the frequency (a weighted percentage) for categorical variables. Demographic characteristics and other covariates were described according to whether individuals had diabetes. Urinary PAH content (from low to high concentrations) was separated into quartiles, Q1, Q2, Q3, or Q4. All PAHs were adjusted by urinary creatinine and used as an independent variable and expressed as ng/g Cr. The urinary concentrations of PAH metabolites were obtained from the NHANES laboratory data files. For concentrations below the Limit of Detection (LOD), we used the values already imputed by NHANES, which employs a standard practice of substituting them with LOD/√2.

In this study, weighted logistic and Cox regression models were used to investigate the individual effects of different urinary PAH metabolites on the prevalence and all-cause mortality in diabetic patients. Three models were constructed to adjust for several confounding variables. Model 1 was unadjusted. Model 2 adjusted for age, sex, race, and PIR. Based on Model 2, Model 3 adjusted for BMI, hypertension, smoking, and alcohol drinking. In addition, possible nonlinear relationships between 6 urinary PAHs measured in logarithmic concentration and outcomes were explored using the Restricted Cubic Spline (RCS). Regarding the relationship between urinary PAH metabolites and diabetes, we calculated the Variance Inflation Factor (VIF) for all independent variables and analyzed the distributions of Pearson residuals and deviance residuals. The relationship between urinary PAH metabolites and all-cause mortality of diabetes was assessed using the Schoenfeld residual test to systematically validate the Cox proportional hazards assumption.

To estimate the combined effects of co-exposed PAH mixtures on diabetes prevalence and all-cause mortality while assessing the contribution of individual PAH compounds, we employed a mixture approach based on WQS regression analysis. This method aimed to evaluate the combined effects and discrete effects of multiple prediction variables in the context of high-dimensional related mixtures. In this analysis, we employed bootstrapping with 10,000 iterations to generate the WQS index. Given that previous studies demonstrated the adverse health effects of PAH exposure, we hypothesized that PAH would be positively correlated with diabetes and related mortality. Therefore, only positive associations were tested in the model. When the WQS index demonstrated statistical significance, we further analyzed the weight of each PAH compound within the index to explore its specific contribution to diabetes prevalence and all-cause mortality. To ensure model robustness, we randomly split the dataset, allocating 40% to the training set and 60% to the validation set.

In the sensitivity analysis, individuals meeting either criterion ‒ self-reported diabetes (“Have you ever been told by a doctor or other healthcare professional that you have diabetes?”) or laboratory criteria of HbA1c ≥ 6.5% or FPG ≥ 126 mg/dL ‒ were defined as having diabetes. Based on this definition, we repeated the WQS analysis. The log-transformed PAH concentrations were incorporated as continuous variables into the fully adjusted Model 3 to further assess the robustness of the results.

All statistical analyses were conducted using R 4.0.3 with the significance level set as two-sided p = 0.05.

## Results

### Characteristics of participants

Baseline characteristics of participants are detailed in Table 1. This study involved 8124 participants, with a mean age of 45-years. Among them, non-diabetic people were aged 44-years on average, and diabetic people were aged 59-years on average. In this study, males accounted for 49%. There were 496 diabetic patients in the male population, slightly greater than that in females.

**Table 1:** Demographic and clinical characteristics of patients with diabetes.

Characteristic	Total number	Overall	NO diabetes	Diabetes	p-value
**Age, years, mean±SE**	8,124	45 (32, 58)	44 (31, 56)	59 (51, 68)	<0.001
**Sex, n (%)**	8,124				0.400
female		4,098 (51%)	3,657 (51%)	441 (49%)	
male		4,026 (49%)	3,530 (49%)	496 (51%)	
**Smoking status, n (%)**	8,124	3,757 (47%)	3,271 (46%)	486(52%)	0.015
**Alcohol drinking status, n (%)**	8,124	5,880 (77%)	5,282 (78%)	598 (66%)	<0.001
**Hypertension, n (%)**	8,124	2,753 (30%)	2,111(27%)	642 (69%)	
**Body mass index (kg/m^2^), n (%)**	8,124				<0.001
Normal (18.5 to < 25)		2,374 (31%)	2,243 (32%)	131 (12%)	
Obese (30 or greater)		2,931 (35%)	2,378 (32%)	553 (63%)	
Overweight (25 to < 30)		2,686 (33%)	2,437 (34%)	249 (25%)	
Underweight (< 18.5)		133 (1.6%)	129 (1.7%)	4 (0.6%)	
**Race, n (%)**	8,124			NA	<0.001
Mexican American		1,294 (8.0%)	1,126 (8.1%)	168 (7.7%)	
Non-Hispanic Black		1,693 (11%)	1,433 (11%)	260 (15%)	
Non-Hispanic White		3,847 (70%)	3,473 (70%)	374 (67%)	
Other Hispanic		618 (4.6%)	541 (4.6%)	77 (4.5%)	
Other Race		672 (6.4%)	614 (6.5%)	58 (5.1%)	
**Poverty-income ratio strata, n (%)**	8,124				0.002
< 1.3		2,560 (21%)	2,216 (21%)	344 (25%)	
> 3.5		2,611 (43%)	2,372 (44%)	239 (36%)	
1.3‒3.5		2,953 (35%)	2,599 (35%)	354 (39%)	

Sociodemographic and health characteristics of participants in the NHANES database, 2003‒2014.

### Associations among PAHs

The heatmap for pairwise associations between urinary PAHs is shown in Fig. S1. Pearson association analysis showed the strongest association between 2-hydroxynaphthalene and 3-hydroxyfluorene (r = 0.86).

### Association of urinary PAHs with the prevalence of diabetes

Urinary PAH exposure showed nominal positive associations with the prevalence of diabetes (Table 2). In Model 1 without covariate adjustment, ΣPAHs (OR = 1.404, 95% CI = 1.063–1.855, p = 0.017) and 2-hydroxynaphthalene (OR = 1.465, 95% CI = 1.137–1.889, p = 0.004) were associated with higher prevalence. In the fully adjusted Model 3, both ΣPAHs (OR = 1.448, 95% CI = 1.073–2.160, p = 0.023) and 1-hydroxynaphthalene (OR = 1.39, 95% CI = 1.040–1.857, p = 0.027) remained positively associated (Figs. 2 and S2). Model diagnostics showed that the VIF for all variables was less than 5, with random distribution of residuals and no outliers, further validating the model's favorable fit (Fig. S3).

**Table 2:** Associations of urinary polycyclic aromatic hydrocarbon metabolites with the prevalence of diabetes.

		Model 1		Model 2		Model 3	
		OR (95% CL)	p	OR (95% CL)	p	OR (95% CL)	p
**ΣPAHs**	Q1 (n = 2031)	Ref		Ref		Ref	
	Q2 (n = 2030)	1.114 (0.840, 1.477)	0.449	1.080 (0.812, 1.436)	0.593	1.102 (0.825, 1.472)	0.506
	Q3 (n = 2030)	1.304 (1.027, 1.654)	0.030	1.223 (0.955, 1.565)	0.109	1.224 (0.950, 1.579)	0.117
	Q4 (n = 2031)	1.404 (1.063, 1.855)	0.017	1.432 (1.063, 1.931)	0.019	1.448 (1.054, 1.988)	0.023
	p for trend	0.007		0.011		0.016	
**1-Hydroxynaphthalene**	Q1 (n = 2031)	Ref		Ref		Ref	
	Q2 (n = 2030)	0.845 (0.609, 1.172)	0.308	0.967 (0.695, 1.346)	0.841	1.000 (0.714, 1.401)	0.999
	Q3 (n = 2030)	0.805 (0.614, 1.054)	0.114	0.970 (0.735, 1.280)	0.828	1.025 (0.767, 1.370)	0.866
	Q4 (n = 2031)	1.071 (0.826, 1.388)	0.601	1.357 (1.028, 1.791)	0.032	1.390 (1.040, 1.857)	0.027
	p for trend	0.611		0.039		0.035	
**2-Hydroxynaphthalene**	Q1 (n = 2031)	Ref		Ref		Ref	
	Q2 (n = 2030)	1.191 (0.925, 1.533)	0.172	1.081 (0.839, 1.391)	0.543	1.084 (0.842, 1.397)	0.527
	Q3 (n = 2030)	1.227 (0.928, 1.623)	0.150	1.047 (0.792, 1.384)	0.745	1.048 (0.785, 1.399)	0.748
	Q4 (n = 2031)	1.465 (1.137, 1.889)	0.004	1.325 (1.006, 1.744)	0.045	1.316 (0.971, 1.784)	0.076
	p for trend	0.006			0.080	0.127	
**3-Hydroxyfluorene**	Q1 (n = 2030)	Ref		Ref		Ref	
	Q2 (n = 2027)	1.023 (0.771, 1.358)	0.871	1.173 (0.885, 1.553)	0.263	1.194 (0.899, 1.585)	0.217
	Q3 (n = 2034)	0.889 (0.703, 1.124)	0.323	0.993 (0.774, 1.274)	0.954	1.026 (0.795, 1.323)	0.843
	Q4 (n = 2031)	1.035 (0.834, 1.283)	0.754	1.219 (0.960, 1.548)	0.104	1.235 (0.920, 1.657)	0.158
	p for trend	0.855		0.311		0.320	
**2-Hydroxyfluorene**	Q1 (n = 2031)	Ref		Ref		Ref	
	Q2 (n = 2030)	0.925 (0.715, 1.196)	0.548	0.967 (0.740, 1.262)	0.800	0.945 (0.726, 1.231)	0.671
	Q3 (n = 2030)	0.936 (0.739, 1.186)	0.581	0.893 (0.702, 1.136)	0.353	0.886 (0.695, 1.129)	0.322
	Q4 (n = 2031)	1.151 (0.903, 1.467)	0.253	1.235 (0.961, 1.588)	0.098	1.246 (0.923, 1.683)	0.149
	p for trend	0.291		0.225		0.322	
**1-Hydroxyphenanthrene**	Q1 (n = 2031)	Ref		Ref		Ref	
	Q2 (n = 2030)	0.714 (0.530, 0.962)	0.027	0.760 (0.560, 1.031)	0.077	0.773 (0.569, 1.050)	0.098
	Q3 (n = 2030)	0.959 (0.736, 1.250)	0.755	1.032 (0.792, 1.344)	0.815	1.029 (0.787, 1.344)	0.834
	Q4 (n = 2031)	0.771 (0.599, 0.992)	0.043	0.914 (0.700, 1.194)	0.506	0.932 (0.705, 1.230)	0.614
	p for trend	0.235		0.904		0.842	
**1-Hydroxypyrene**	Q1 (n = 2031)	Ref		Ref		Ref	
	Q2 (n = 2030)	0.831 (0.633, 1.091)	0.181	0.807 (0.612, 1.065)	0.129	0.794 (0.599, 1.054)	0.109
	Q3 (n = 2030)	0.947 (0.727, 1.233)	0.682	0.941 (0.723, 1.226)	0.650	0.951 (0.724, 1.250)	0.717
	Q4 (n = 2031)	0.926 (0.713, 1.202)	0.559	1.021 (0.773, 1.349)	0.884	1.038 (0.777, 1.386)	0.798
	p for trend	0.741		0.742		0.652	

Model 1: Unadjusted. Model 2: Adjusted for age, sex, race, and income. Model 3: Adjusted for age, sex, race, income, BMI, hypertension, smoking, and alcohol consumption.

Note: Nominal p-values are shown. Results adjusted for multiple comparisons (FDR) are provided in Supplementary Table S3.

**Fig. 2:**
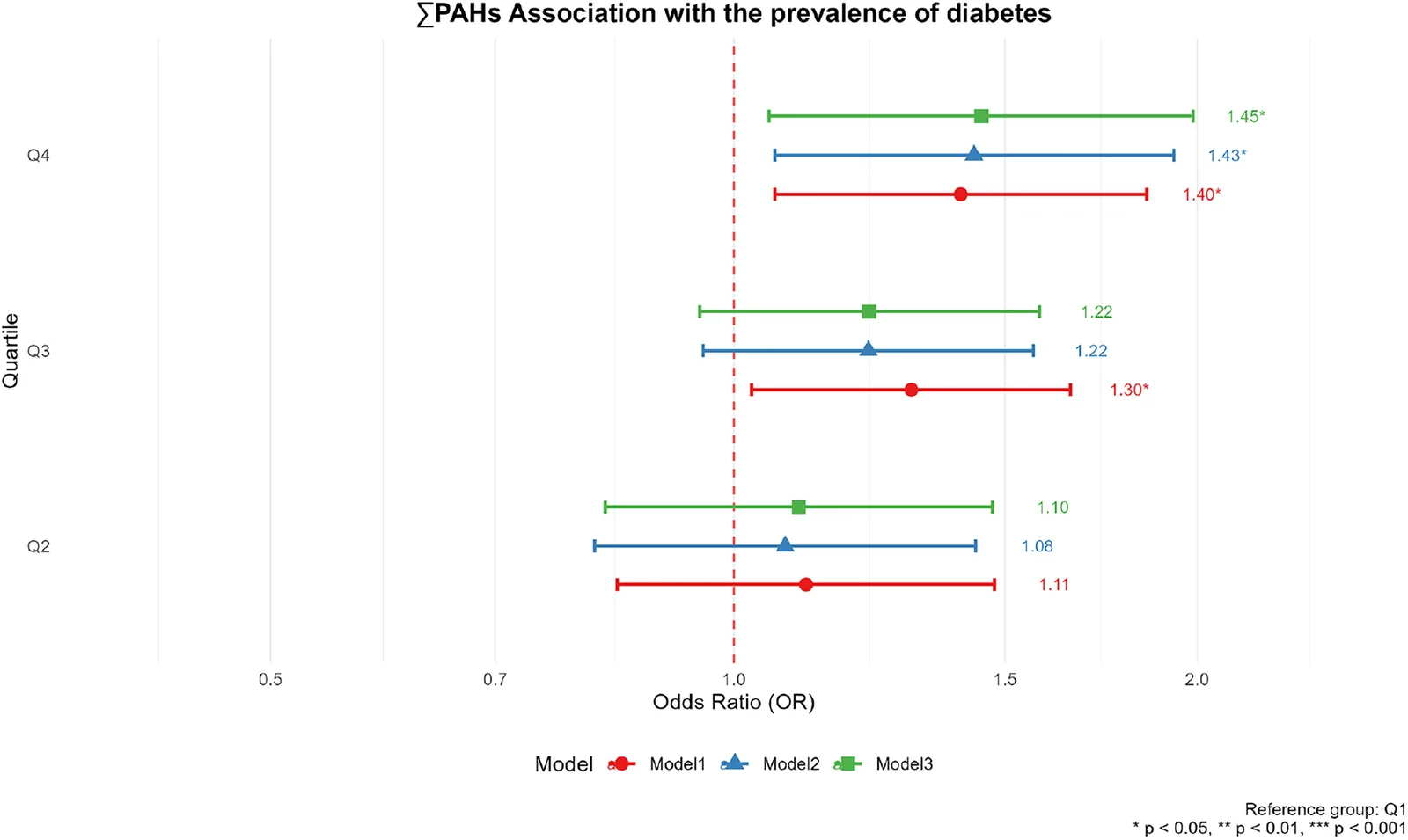
Forest plot of the association between ΣPAHs and diabetes prevalence.

### Association of urinary PAHs with all-cause mortality in diabetic patients

To further explore the hazards to humans posed by PAHs, Cox regression analysis was performed to explore the association between urinary PAHs and all-cause mortality of diabetes (Table 3). When no covariates were adjusted (Model 1), 2-hydroxynaphthalene was associated with a 52% increase in all-cause mortality in Q4 compared to Q1 (HR = 1.526, 95% CI = 1.052, 2.214, p = 0.026). Although no significant correlation trend was found in the regression analysis of individual PAHs, a nominal positive association was observed for ΣPAHs and all-cause mortality (Fig. 3). In Model 1, ΣPAHs demonstrated a 52% increase in all-cause mortality in Q4 compared to Q1 (HR = 1.523, 95% CI = 1.073, 2.160, p = 0.018). After adjustment for age, sex, race, and PIR (Model 2), ΣPAHs exhibited a 44% increase in all-cause mortality in Q4 compared to Q1 (HR = 1.442, 95% CI = 1.001, 2.078, p = 0.049). Notably, the increase in mortality risk was modest, and the association only reached borderline statistical significance (p = 0.049) (Fig. S4). The Schoenfeld residuals test was used to validate the Cox proportional hazards assumption. For the primary exposure variable (∑PAHs), the p-value for all covariates was > 0.05 (p = 0.2266) (Fig. S5).

**Table 3:** Associations of urinary polycyclic aromatic hydrocarbon metabolites with all-cause mortality in diabetes.

		Model 1		Model 2		Model3	
		HR (95% CL)	p	HR (95% CL)	p	HR (95% CL)	p
**ΣPAHs**	Q1 (n = 2031)	Ref		Ref		Ref	
	Q2 (n = 2030)	1.060 (0.698, 1.608)	0.786	1.051 (0.681, 1.623)	0.821	1.026 (0.668, 1.575)	0.907
	Q3 (n = 2030)	1.227 (0.839, 1.795)	0.291	1.160 (0.791, 1.701)	0.447	1.121 (0.757, 1.660)	0.570
	Q4 (n = 2031)	1.523 (1.073, 2.160)	0.018	1.442 (1.001, 2.078)	0.049	1.320 (0.902, 1.931)	0.153
	p for trend	0.009		0.030		0.109	
**1-Hydroxynaphthalene**	Q1 (n = 2031)	Ref		Ref		Ref	
	Q2 (n = 2030)	0.969 (0.580, 1.620)	0.905	1.045 (0.615, 1.774)	0.871	0.972 (0.568, 1.664)	0.917
	Q3 (n = 2030)	0.755 (0.445, 1.282)	0.298	0.730 (0.420, 1.267)	0.263	0.713 (0.406, 1.253)	0.240
	Q4 (n = 2031)	1.283 (0.864, 1.903)	0.216	1.226 (0.784, 1.918)	0.372	1.112 (0.687, 1.799)	0.665
	p for trend	0.276		0.501		0.774	
**2-Hydroxynaphthalene**	Q1 (n = 2031)	Ref		Ref		Ref	
	Q2 (n = 2030)	0.898 (0.627, 1.286)	0.557	0.829 (0.563, 1.219)	0.340	0.849 (0.578, 1.249)	0.406
	Q3 (n = 2030)	0.993 (0.678, 1.453)	0.969	0.999 (0.687, 1.452)	0.995	0.994 (0.683, 1.447)	0.975
	Q4 (n = 2031)	1.526 (1.052, 2.214)	0.026	1.447 (0.959, 2.183)	0.078	1.321 (0.888, 1.963)	0.169
	p for trend	0.037		0.063		0.146	
**3-Hydroxyfluorene**	Q1 (n = 2030)	Ref		Ref		Ref	
	Q2 (n = 2027)	0.878 (0.588, 1.310)	0.524	0.768 (0.510, 1.157)	0.207	0.746 (0.500, 1.114)	0.153
	Q3 (n = 2034)	0.836 (0.542, 1.288)	0.416	0.768 (0.493, 1.196)	0.243	0.738 (0.470, 1.161)	0.189
	Q4 (n = 2031)	1.269 (0.808, 1.993)	0.300	1.039 (0.641, 1.686)	0.876	0.888 (0.539, 1.462)	0.641
	p for trend	0.601		0.889		0.491	
**2-Hydroxyfluorene**	Q1 (n = 2031)	Ref		Ref		Ref	
	Q2 (n = 2030)	1.010 (0.650, 1.569)	0.964	0.950 (0.616, 1.463)	0.815	0.904 (0.582, 1.404)	0.652
	Q3 (n = 2030)	0.983 (0.592, 1.633)	0.948	0.919 (0.536, 1.573)	0.757	0.875 (0.514, 1.489)	0.623
	Q4 (n = 2031)	1.389 (0.921, 2.096)	0.117	1.196 (0.768, 1.861)	0.428	1.032 (0.648, 1.642)	0.896
	p for trend	0.202		0.553		0.986	
**1-Hydroxyphenanthrene**	Q1 (n = 2031)	Ref		Ref		Ref	
	Q2 (n = 2030)	0.818 (0.546, 1.227)	0.331	0.813 (0.507, 1.304)	0.390	0.818 (0.520, 1.288)	0.386
	Q3 (n = 2030)	0.819 (0.557, 1.204)	0.310	0.828 (0.540, 1.269)	0.386	0.760 (0.492, 1.173)	0.216
	Q4 (n = 2031)	0.758 (0.503, 1.142)	0.185	0.719 (0.466, 1.111)	0.137	0.647 (0.416, 1.005)	0.053
	p for trend	0.193		0.165		0.056	
**1-Hydroxypyrene**	Q1 (n = 2031)	Ref		Ref		Ref	
	Q2 (n = 2030)	0.769 (0.533, 1.110)	0.161	0.730 (0.508, 1.050)	0.089	0.642 (0.443, 0.931)	0.020
	Q3 (n = 2030)	0.625 (0.419, 0.934)	0.022	0.609 (0.409, 0.907)	0.015	0.520 (0.337, 0.802)	0.003
	Q4 (n = 2031)	1.157 (0.729, 1.834)	0.536	1.022 (0.657, 1.589)	0.922	0.874 (0.549, 1.391)	0.569
	p for trend	0.625		0.359		0.114	

Model 1: Unadjusted. Model 2: Adjusted for age, sex, race, and income. Model 3: Adjusted for age, sex, race, income, BMI, hypertension, smoking, and alcohol consumption.

Nominal p-values are shown. Results adjusted for multiple comparisons (FDR) are provided in Supplementary Table S3.

**Fig. 3:**
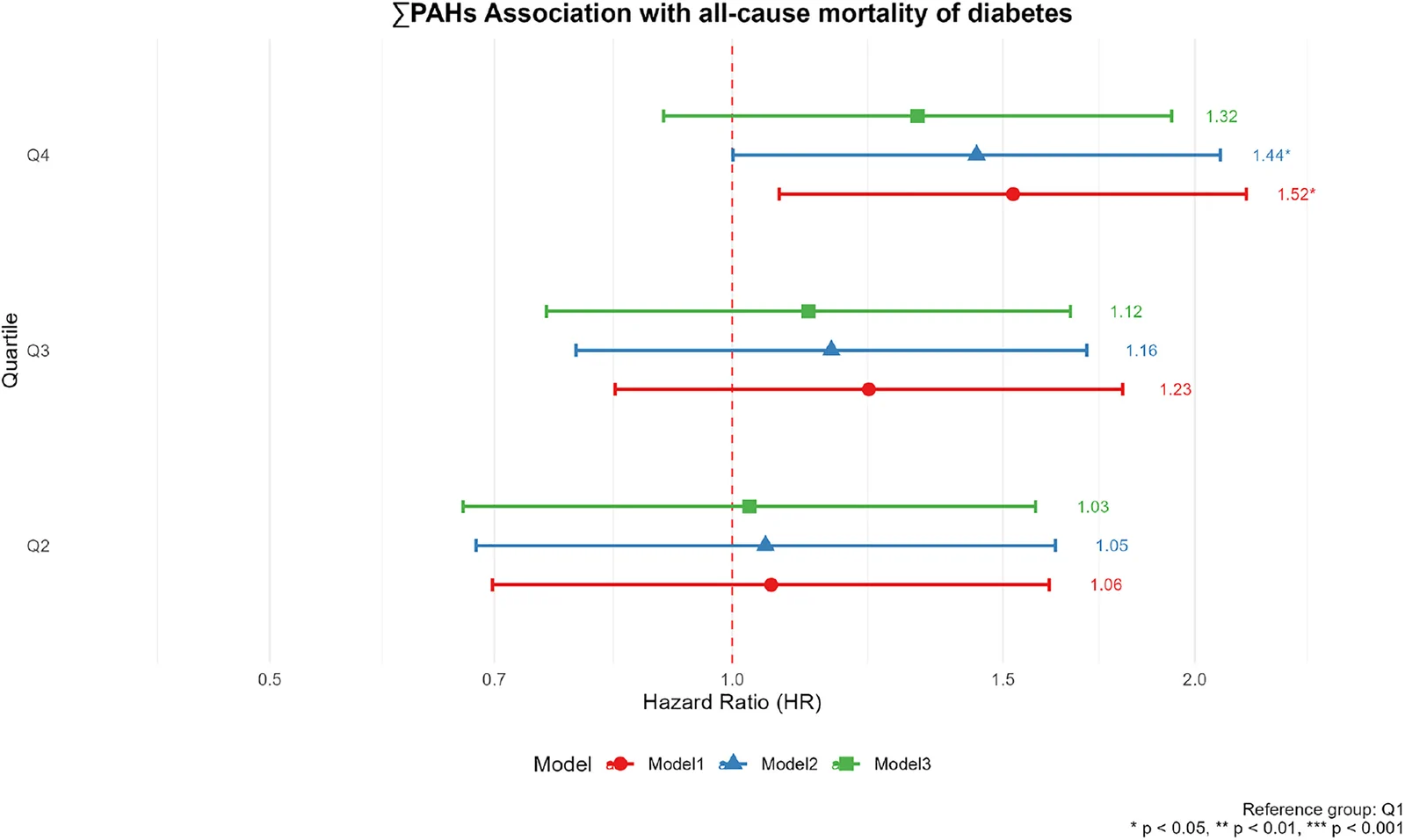
Forest plot of the association between ΣPAHs and all-cause mortality in diabetes.

RCS analysis (Fig. 4, Fig. 5) revealed linear relationships between the log-transformed concentrations of all six PAHs and the prevalence of diabetes. For the all-cause mortality of diabetes, only the log-transformed concentration of 2-hydroxynaphthalene had a nonlinear relationship with it, while the log-transformed concentrations of the other five PAHs showed a linear relationship with it.

**Fig. 4:**
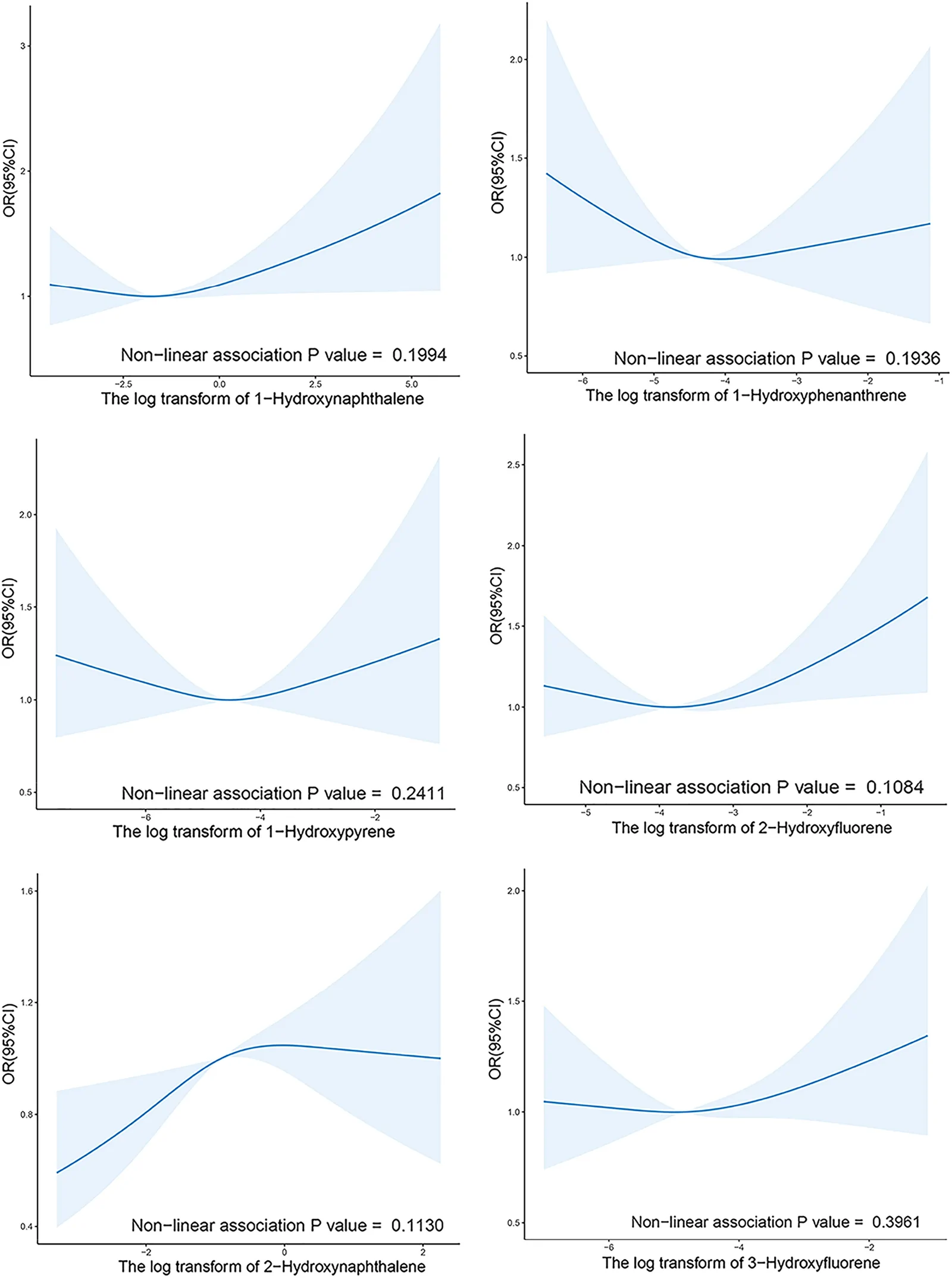
Exposure-response relationships between adjusted log-transformed urinary polycyclic aromatic hydrocarbon concentrations and diabetes prevalence. (Shaded area represents the 95% Confidence Interval).

**Fig. 5:**
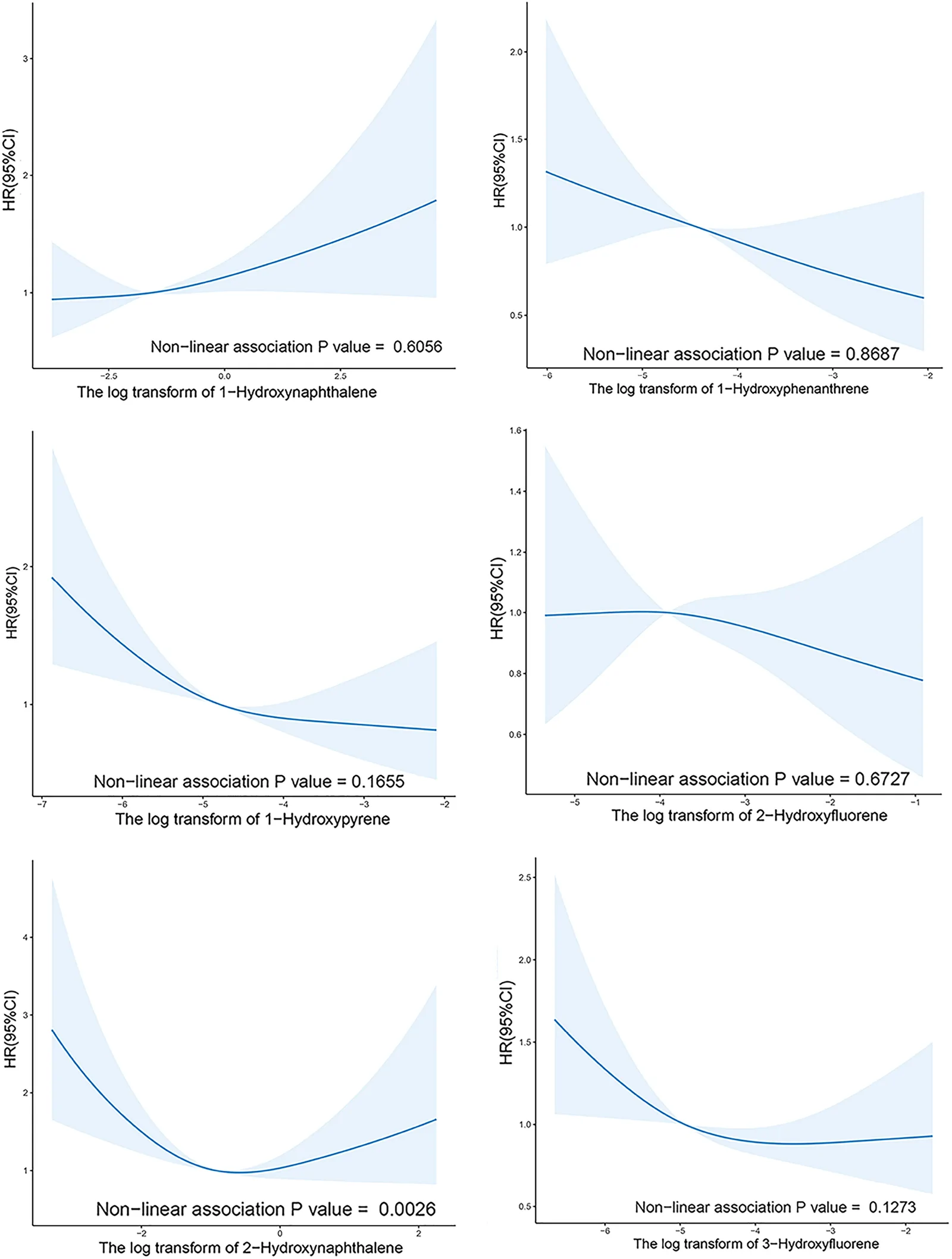
Exposure-response relationships between adjusted log-transformed urinary polycyclic aromatic hydrocarbon concentrations and diabetes all-cause mortality.

### WQS regression analysis

The WQS regression model found that the content of the PAH mixture was significantly positively associated with both the prevalence (OR = 1.131, 95% CI = 1.008, 1.269, p = 0.03) and the all-cause mortality (OR = 1.33, 95% CI = 1.051, 1.684, p = 0.01) of diabetes (Fig. 6). In addition, 2-hydroxynaphthalene had the greatest effect on the prevalence of diabetes, and 1-hydroxynaphthalene had the greatest effect on the all-cause mortality of diabetes. The exact mean weights of all chemical components included in this study are provided in Table S1.

**Fig. 6:**
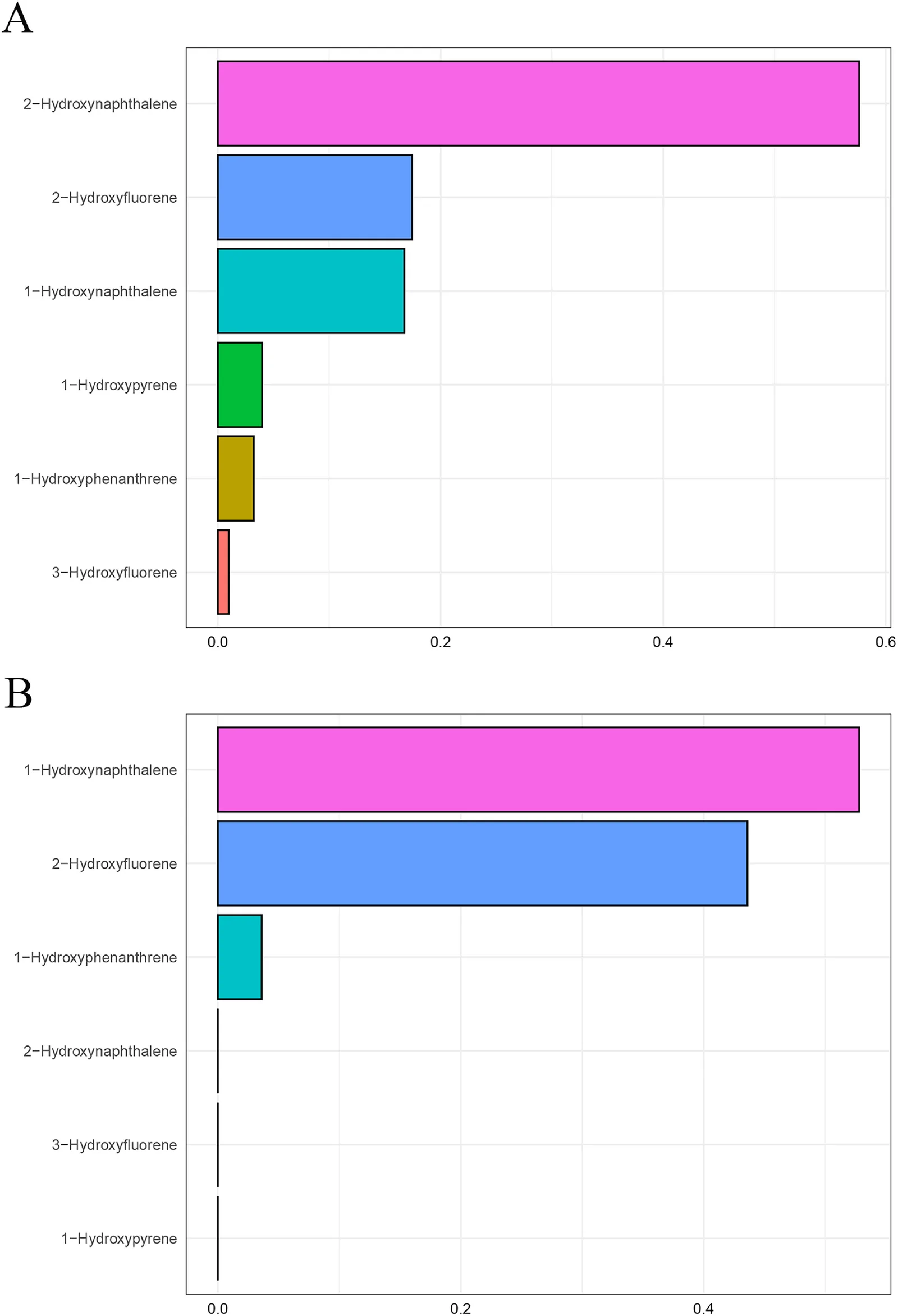
(A) Associations of urinary polycyclic aromatic hydrocarbon metabolites with the prevalence of diabetes; (B) Associations of urinary polycyclic aromatic hydrocarbon metabolites with all-cause mortality in diabetes.

### Sensitivity analysis

Diabetes was redefined by self-reported diabetes and laboratory criteria. Under this broader definition, the number of participants with diabetes increased from 936 to 941, and the overall sample size for this analysis was 8,298. WQS regression analysis revealed that the effect of exposure to PAH mixtures remained significant based on this definition. PAH mixtures maintained a significant positive association with diabetes prevalence (OR = 1.136, 95% CI = 1.013–1.274, p = 0.029) and were also significantly associated with the risk of all-cause mortality from diabetes (HR = 1.352, 95% CI = 1.048–1.677, p = 0.019). To further enhance the interpretability of the results, we analyzed the log-transformed PAH concentrations as continuous variables. Results indicated that the association between 1-Hydroxynaphthalene and diabetes remained significant (OR = 1.075, 95% CI = 1.010, 1.143, p = 0.023), consistent with the quartile analysis. However, when examining the relationship between PAH and all-cause mortality from diabetes, the continuous variable did not reach significance (p > 0.05). Nevertheless, the continuous exposure model still demonstrated a positive correlation trend consistent with the quartile model (Table S2).

## Discussion

In this study, logistic regression, Cox analysis, and WQS models were employed to systematically investigate the associations between single and mixed exposure effects of PAHs and the prevalence of diabetes as well as all-cause mortality. WQS regression revealed associations between mixed exposure to six PAHs and both diabetes prevalence and all-cause mortality, with 2-hydroxynaphthalene contributing most to diabetes risk and 1-hydroxynaphthalene to mortality risk within the mixture. In exploratory analyses of individual metabolites, ΣPAHs, 1-hydroxynaphthalene, and 2-hydroxynaphthalene showed nominal positive associations with diabetes prevalence, while ΣPAHs and 2-hydroxynaphthalene exhibited modest associations with all-cause mortality.

WQS analysis found that 2-hydroxynaphthalene significantly influenced the prevalence of diabetes, consistent with previous research. A case-control study involving 147 patients with type 1 diabetes further validated the association between 2-hydroxynaphthalene and diabetes^12^ The study found that individuals with higher urinary concentrations of 2-hydroxynaphthalene and naphthalene metabolites had a significantly higher risk of diabetes than those with lower concentrations. This suggests that different types of diabetes may share common environmental risk factors. These findings underscore the significance of 2-hydroxynaphthalene as a potential biomarker. Furthermore, we identified for the first time that 1-hydroxynaphthalene exerted the most pronounced effect on all-cause mortality in diabetes, revealing the intricate relationship between PAH metabolites and disease-specific mortality. Existing literature has generally confirmed the significant association between PAH metabolites and all-cause mortality^13^ In particular, 1-hydroxynaphthalene and 2-hydroxynaphthalene are positively correlated with both all-cause mortality and cardiovascular mortality^14^ Research also indicates that increased PAH concentrations are significantly associated with an elevated risk of mortality, with an increased mortality risk observed even at extremely low levels of short-term exposure^15^ Our study fills a gap in understanding the relationship between 1-hydroxynaphthalene and all-cause mortality in diabetes, highlighting its role as a key metabolite. Future research should further investigate the role of PAHs and their metabolites in the development and progression of diabetes, and explore strategies to reduce environmental exposure to lower the risk of diabetes-related mortality.

PAH exposure is closely associated with elevated incidence and mortality rates of diabetes. Its core mechanisms primarily involve inflammatory responses, oxidative stress, and insulin resistance. PAHs can activate the Aryl Hydrocarbon Receptor (AhR), induce the expression of Cytochrome P450 enzymes (CYP1A1/1B1), accelerate PAH metabolism, and generate large amounts of Reactive Oxygen Species (ROS), thereby triggering oxidative stress^16^^,^^17^ This oxidative stress disrupts the functional structure of pancreatic β-cells, induces the release of pro-inflammatory factors (such as TGF-β, IL-1β), exacerbates inflammatory responses, and ultimately leads to β-cell damage, impaired insulin secretion, and insulin resistance ‒ serving as a key driver of diabetes development.^18^, ^19^, ^20^

IL-22 plays a crucial protective role in this process. Studies have found that PAHs exposure significantly reduces IL-22 levels,^21^ impairing its protective capacity on β-cells and exacerbating ROS-induced apoptosis. Oxidative stress and inflammatory responses triggered by AhR activation jointly inhibit IL-22 expression, thereby forming the pathogenic pathway: “PAHs-AhR-ROS/inflammatory response-IL-22 downregulation-β-cell damage”^21^^,^^22^

Furthermore, PAHs-induced oxidative stress also promotes diabetes progression through epigenetic mechanisms. For instance, it can cause abnormal DNA methylation in β-cells, inhibit the expression of key transcription factors (such as PDX1, ARX), and affect the development and functional maintenance of β-cells^23^^,^^24^ Such epigenetic alterations may exert long-term effects on the expression of genes related to glucose metabolism, thereby aggravating the diabetic condition^25^^,^^26^ (Fig. 7).

**Fig. 7:**
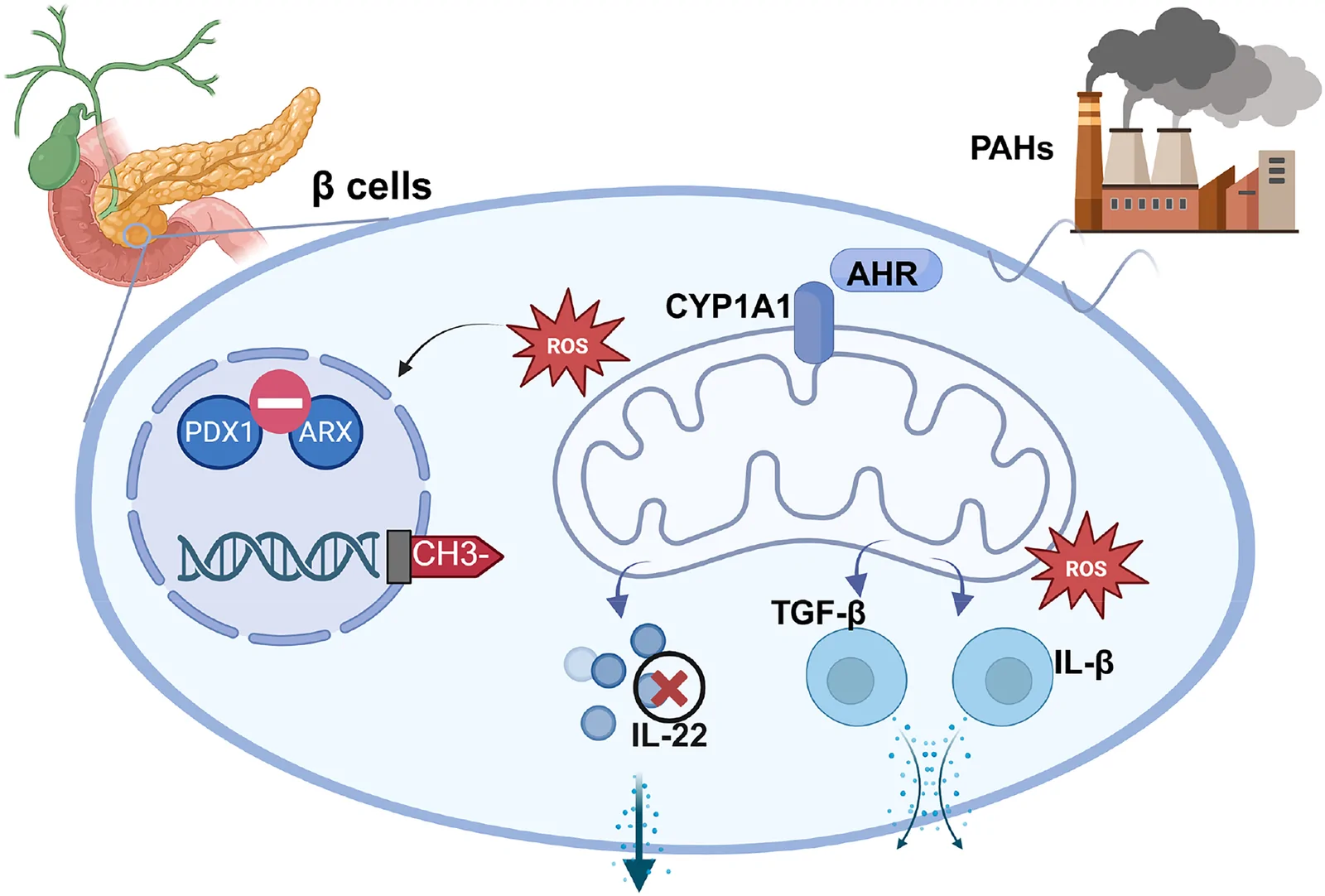
Mechanistic diagram of the relationship between polycyclic aromatic hydrocarbons and diabetes. Created in BioRender. li, a. (2025) https://BioRender.com/y60ahiv.

The WQS regression method has been widely applied in numerous studies to analyze the combined effects of mixed exposure to multiple environmental pollutants on disease outcomes. By calculating the WQS index, this approach enables the assessment of how a set of exposures influences disease progression and reveals the role of each pollutant within the mixed exposure. Previous research has validated the model's effectiveness in evaluating the association between PAHs and disease. For example, in studies examining the relationship between PAHs and hypertension, WQS regression analysis identified naphthalene metabolites as the primary factor influencing systolic blood pressure^27^ Regarding the relationship between PAHs and diabetes prevalence, traditional linear regression models are commonly used to analyze the association between biomarkers and PAH exposure^3^ However, this approach fails to compare the risk factors of individual components within mixed exposures. Therefore, the WQS model helps gain a more comprehensive understanding of the interactions among different PAH metabolites and their combined impact on diabetes. We employed WQS regression to investigate the effects of six PAH compounds on diabetes. Results indicated that the combined effects of these PAH compounds were significantly positively correlated with diabetes prevalence and all-cause mortality. Among them, 2-hydroxynaphthalene exerted the strongest influence on diabetes prevalence, while 1-hydroxynaphthalene had the strongest association with diabetes-related all-cause mortality. This suggests that the overall effect of PAHs may be mediated by specific metabolites, while other metabolites exert distinct roles in different aspects of diabetes, such as mortality or prevalence. However, WQS still has several limitations. WQS regression assumes same direction of effects; thus, if some mixture components act in opposite directions, the estimated overall mixture effect may be biased. Moreover, WQS regression may not fully capture potential non-linear associations or interactions among mixture components. Future studies can use more flexible mixture modeling approaches, such as QGcomp or BKMR, to validate and extend our findings.

Certain limitations should be noted in this investigation. First, although cross-sectional data reveal an association between PAHs and diabetes, they do not establish causality. Future long-term cohort studies could validate it. Second, while multiple demographic and behavioral covariates were accounted for, we cannot rule out potential residual covariates, such as diet, physical activity, and occupational exposure, which may influence the results. Although this study assessed PAH metabolite levels through a single urine measurement, this method reflected only recent exposure due to the short half-life of PAH metabolites and may not accurately represent long-term exposure. Furthermore, PAH metabolism and excretion can vary significantly between individuals, influenced by age, sex, and muscle mass. Older individuals or those with lower muscle mass may exhibit different metabolic rates, leading to variations in metabolite concentrations under identical PAH exposure conditions. Future research should consider employing repeated measurements or other biomarkers (such as PAH levels in blood or hair) to better assess long-term exposure and elucidate the causal relationship between PAHs and chronic diseases. Third, due to the lack of urinary PAH metabolite data, a large number of participants were excluded, substantially reducing the final analytic sample. This missingness mainly stems from the NHANES laboratory testing protocol rather than individual participant characteristics, which partially mitigates the risk of selection bias. However, the final sample only represents individuals who completed comprehensive physical examinations and provided valid biospecimens, so the findings may not be fully generalizable to the broader U.S. adult population. Further studies are warranted to confirm these findings in broader, more representative samples. Finally, after FDR correction, none of the associations between individual PAH metabolites and diabetes prevalence or all-cause mortality remained statistically significant (Table S3). Therefore, these nominal associations should be interpreted as exploratory. Although FDR correction may be conservative due to correlations among PAH metabolites, we reported the corrected results to reduce the risk of false-positive findings. Thus, the main conclusions of this study should be interpreted primarily on the mixture exposure models.

## Conclusion

Our study found a link between co-exposure to six urinary Polycyclic Aromatic Hydrocarbons (PAHs) and higher diabetes prevalence and all-cause mortality. Specifically, 2-hydroxynaphthalene contributed most to diabetes risk, while 1-hydroxynaphthalene was associated with mortality risk. These findings highlight the potential health risks of urinary PAH exposure, necessitating further research to elucidate the causal mechanisms and identify effective public health interventions to mitigate PAH exposure.

## Ethics approval and consent to participate

Not applicable.

## Consent for publication

Not applicable.

## AI-assisted technologies statement

The authors did not use any Artificial Intelligence (AI) or AI-assisted technologies in the creation of this manuscript.

## Authors’ contributions

All authors contributed to the study conception and design. Writing-original draft preparation: Ao Li, Yuxin Pan, Mingcong Chen, Yang Meng, Yang Zhang; Writing-review and editing: Ao Li, Mingyi Zhao, Lin Liu; Conceptualization: Ao Li, Lin Liu; Methodology: Ao Li, Yuxin Pan, Mingcong Chen, Yang Meng, Yang Zhang; Formal analysis and investigation: Yuxin Pan, Mingcong Chen, Yang Meng, Yang Zhang; Funding acquisition: Mingyi Zhao, Lin Liu; Resources: Mingyi Zhao, Lin Liu; Supervision: Mingyi Zhao, Lin Liu, and all authors commented on previous versions of the manuscript. All authors read and approved the final manuscript.

## Funding

This work was supported by the 10.13039/100014717Youth Project of National Natural Science Foundation of China (82102280) and 10.13039/501100004735Natural Science Foundation of Hunan Province (2022JJ30893) and Talent Attraction and Cultivation Foundation (YX202212).

## Conflicts of interest

The authors declare no have conflicts of interest.

## Data Availability

The data of this study were all derived from the NHANES database.
