# Supplementary material for: Association between urinary polycyclic aromatic hydrocarbon metabolites and diabetes-related mortality: a longitudinal study in U.S. adults
Source: Clinics (Sao Paulo). 2026 Sep 29;81:101169. doi: 10.1016/j.clinsp.2026.101169 (PMC13653480; doi:10.1016/j.clinsp.2026.101169)
Supplement: Supplementary file 1 [file mmc1.docx]

**CLINICS-D-25-01541**

**Supplementary Materials**

**Table S1** Mean weights of PAH metabolites in the WQS regression models for diabetes and mortality.

| **Mixture component** | **Mean weight (Diabetes)** | **Mean weight (Mortality)** |
| --- | --- | --- |
| 2-Hydroxynaphthalene | 0.64041708746 | 0.03309043458 |
| 1-Hydroxynaphthalene | 0.20628645919 | 0.45383579112 |
| 3-Hydroxyfluorene | 0.12538387574 | 0.00000000433 |
| 1-Hydroxypyrene | 0.02327741662 | 0.00000000464 |
| 2-Hydroxyfluorene | 0.00456478912 | 0.35329068356 |
| 1-Hydroxyphenanthrene | 0.00007037187 | 0.15978308176 |

**Table S2** (A) Association between log-transformed PAH and prevalence of diabetes in Model 3; (B) Association between log-transformed PAH and all-cause mortality in Model 3.

| **(A)** | **Prevalence of Diabetes (Model 3)** | |
| --- | --- | --- |
|  | **OR (95% CL)** | **p** |
| log(ΣPAHs) | 1.108 (0.997, 1.083) | 0.088 |
| log(1-Hydroxynaphthalene) | 1.075 (1.010, 1.143) | 0.023 |
| log(2-Hydroxynaphthalene) | 1.090 (0.974, 1.220) | 0.133 |
| log(3-Hydroxyfluorene) | 1.048 (0.964, 1.140) | 0.267 |
| log(2-Hydroxyfluorene) | 1.079 (0.971, 1.199) | 0.155 |
| log(1-Hydroxyphenanthrene) | 0.986 (0.874, 1.113) | 0.819 |
| log(1-Hydroxypyrene) | 1.042 (0.937, 1.158) | 0.445 |
| **(B)** | **All-cause mortality in Diabetes (Model 3)** | |
|  | **HR (95% CL)** | **p** |
| log(ΣPAHs) | 1.001 (0.970, 1.032) | 0.9 |
| log(1-Hydroxynaphthalene) | 1.054 (0.938, 1.184) | 0.4 |
| log(2-Hydroxynaphthalene) | 1.104 (0.974, 1.251) | 0.12 |
| log(3-Hydroxyfluorene) | 0.963 (0.830, 1.116) | 0.6 |
| log(2-Hydroxyfluorene) | 1.026 (0.891, 1.182) | 0.7 |
| log(1-Hydroxyphenanthrene) | 0.790 (0.652, 0.957) | 0.016 |
| log(1-Hydroxypyrene) | 0.920 (0.779, 1.087) | 0.3 |

Model 3: Adjusted for age, sex, race, income, BMI, hypertension, smoking, and alcohol consumption.

**Table S3** Raw and FDR-adjusted p-values for PAH metabolites associations with diabetes prevalence and mortality.

| **PAH** | **Prevalence_Original_P** | **Prevalence_FDR_P** | **Mortality_Original_P** | **Mortality_FDR_P** |
| --- | --- | --- | --- | --- |
| ∑PAHs | 0.023 | 0.0945 | 0.153 | 0.394 |
| 1-hydroxynaphthalene | 0.027 | 0.0945 | 0.665 | 0.776 |
| 2-hydroxynaphthalene | 0.076 | 0.177 | 0.169 | 0.394 |
| 3-hydroxyfluorene | 0.158 | 0.2212 | 0.641 | 0.776 |
| 2-hydroxyfluorene | 0.149 | 0.2212 | 0.896 | 0.896 |
| 1-hydroxyphenanthrene | 0.614 | 0.716 | 0.053 | 0.371 |
| 1-hydroxypyrene | 0.798 | 0.798 | 0.569 | 0.776 |

**Figure S1** Spearman correlation coefficients of polycyclic aromatic hydrocarbon metabolites.

**Figure S2** Forest plot of the association between PAH and diabetes prevalence.

**Figure S3** Residuals vs. fitted values scatter plot of polycyclic aromatic hydrocarbon metabolites.

**Figure S4** Forest plot of the association between PAH and all-cause mortality in diabetes.

**Figure S5** Schoenfeld residuals plot of polycyclic aromatic hydrocarbon metabolites.
